# COVID-19 Implications on Clinical Clerkships and the Residency Application Process for Medical Students

**DOI:** 10.7759/cureus.7800

**Published:** 2020-04-23

**Authors:** Allison Akers, Christian Blough, Maya S Iyer

**Affiliations:** 1 Medicine, The Ohio State University Wexner Medical Center, Columbus, USA; 2 Pediatrics, Nationwide Children’s Hospital, Columbus, USA; 3 Pediatrics, The Ohio State University College of Medicine, Columbus, USA

**Keywords:** covid-19, clerkships, residency application, medical education

## Abstract

The coronavirus disease 2019 (COVID-19) pandemic has caused significant disruption to undergraduate medical education (UME). Although the immediate scheduling challenges are being addressed, there has been less discourse regarding how this pandemic will impact medical students in their preparation for and application to residency programs. While some historical disasters and pandemics provide a loose precedent for UME response during COVID-19, the impact of the current pandemic has surpassed any other events. COVID-19 will likely impact UME in the suspension of clinical rotations, alterations in grading, suspension or elimination of away rotations, changes in medical licensing exams, and ramifications on mental health. This review assesses governing medical bodies’ recommendations regarding UME during the COVID-19 pandemic and how this may impact preparation for residency. In particular, residency programs will likely have to create new guidelines for assessing applicants during this unique cycle.

## Introduction and background

The coronavirus disease 2019 (COVID-19) pandemic has led to widespread uncertainty and affected every corner of the globe. In the United States, the pandemic has had profound medical, political, and financial impacts. While much research and discussion surround the immediate and potentially enduring political, financial, and public health ramifications of the COVID-19 pandemic, the dialogue regarding how COVID-19 will affect undergraduate medical education (UME) is somewhat lagging. The current UME discourse has focused on alterations to pre-clinical and clinical education, with limited discussion regarding how this ultimately impacts the residency application process [1-3]. On March 17, 2020, the American Association of Medical Colleges (AAMC) released guidelines recommending a minimum two-week suspension on any clinical activities involving patient contact with the goal of conserving personal protective equipment (PPE), minimizing the potential spread of the virus, and protecting learners [4]. These recommendations were later extended to April 14, 2020, with new guidelines suggesting lengthened suspensions in accordance with local, state, and national guidelines. In addition, the AAMC “strongly suggest[s] that medical students not be involved in any direct patient care activities” [4-5]. This integrative review discusses the impact of the COVID-19 pandemic on the clerkship learning environment, medical licensing examinations, the mental health of medical students, and how these changes will affect the residency application process for medical students graduating in 2021.

## Review

Clinical clerkships

Clinical rotations generally commence in the third year of medical school and are a vital component of UME. While the COVID-19 pandemic has the potential to cause the most disruption to the clerkship learning environment, historical disasters and epidemics do provide a precedent for how medical education can be altered in response to disasters. In 2005, Hurricane Katrina forced the closure of the Tulane University School of Medicine. Communications between Baylor College of Medicine, other universities, the Liaison Committee on Medical Education (LCME), and the AAMC allowed for the remarkable return to clinical clerkships at outside university hospitals within one month of the storm [6]. However, Hurricane Katrina affected medical schools only in New Orleans, while COVID-19 is affecting medical schools and academic hospitals across the country and world. Perhaps a more comparable scenario would be to the severe acute respiratory syndrome (SARS) outbreak of the early 2000s which forced medical school closures in both Hong Kong and Canada [7-8]. In response to canceled clerkships, many medical schools during that time successfully transitioned to videotaped vignettes, audiotaped recordings, or online chat rooms and webcasting to replace clinical experiences [9-10]. While simulation may provide alternate clinically related opportunities for medical schools facing the current COVID-19 pandemic, there is a lack of fidelity and realism with such learning methods. As a result, these medical students will have reduced clinical opportunities prior to applying for residency programs.

The current AAMC guidelines initially outlined a minimum of four weeks suspension of clinical clerkship rotations and have now strongly suggested a prolonged extension of this suspension [4-5]. The LCME has responded to these suspensions by suggesting shortening required clinical clerkships, finding other curricular areas where clerkship objectives may be addressed, and transitioning to virtual learning for certain components of clinical education [11]. However, for many students, this will significantly hinder educational opportunities. Some students may miss the opportunity for a full rotation in a specialty of interest, limiting exposure to various areas of medicine and chances to network and be mentored by specialty faculty. For others, the extended period of time suspended from clerkships will disrupt the ability to grow essential clinical decision-making skills that are necessary to be successful in residency and beyond.

Grading in UME

In both of the aforementioned scenarios, medical students who were removed from clinical clerkships faced significant challenges. In the aftermath of Hurricane Katrina, many students demonstrated a decline in performance on clinical course examinations, potentially due to external stressors [10]. Overwhelming external pressures during COVID-19 may also negatively affect student performance on clinical examinations. Because this pandemic will likely impact academic performance, the LCME has sought to address methods of assessments for clinical clerkships [11]. The LCME suggests the possibility of moving to pass/fail grading for clinical experiences at this phase of the curriculum [11]. However, this leaves the opportunity for national variability in clinical clerkship grading. Additionally, since this pandemic has disproportionately affected certain areas of the country, some medical students may be at risk for a more significant decline in academic performance in clerkship examinations and may suffer from a lack of national uniformity in clinical clerkship assessments. Furthermore, the negative impact on clerkship grades will influence part of the Medical Student Performance Evaluations (MSPEs), which residency programs use as a summary of a student’s experiences, attributes, and academic performance and are meant to provide objective data for residency program directors [12-13]. Residency programs use required clerkship grades, MSPEs, and other factors in determining which applicants receive interviews. This suggests that the COVID-19 pandemic may have enduring implications on the professional careers of many medical students [14].

Away rotations 

Although educators may argue that away rotations are expensive and only partly educational, such clinical experiences are a critical part of the residency application process for many specialties [15-16]. Institutions such as the University of California San Diego School of Medicine and Boston University School of Medicine have already canceled or postponed current and future away rotations [17-18]. Medical students can elect to partake in away rotations for increased educational exposure in a specific specialty, assessment of fit at particular programs, desire for letters of recommendation, and the ability to show interest in a specific geographic region [15]. Survey data from the 2014-2015 Electronic Residency Application Service (ERAS) shows that 67% of fourth-year medical students complete an away rotation [16]. In certain specialties, such as orthopedic surgery and plastic surgery, the percentage of students that match into a residency program at an institution at which they rotated, including both home institution and away rotations is high, 57% and 44% respectively [19-20]. These results suggest that away rotations play a significant role in the residency match process. At particular risk from the suspension or elimination of away rotations are students without a subspecialty rotation at their home institution. For example, in order for medical students applying into otolaryngology, who did not have that specialty at their home institution, to be as competitive as those with a home-based program, they have to do more away rotations [21]. If the current pandemic affects away rotations, the match process will undoubtedly be different, for medical students graduating in 2021, in a way that both applicants and institutions have not previously experienced. Specifically, away rotations are essential in creating accessible opportunities for medical students at institutions without the subspecialty rotations of choice. The elimination of away rotations may create barriers for these students in becoming competitive residency applicants.

Medical licensing examinations

In addition, clinical licensing exams, which are taken during the clinical rotations, have also been affected by the COVID-19 pandemic. The United States Medical Licensing Examinations (USMLE) are essential parts of the progression through medical education and the medical licensing process and are required for application to residency programs. Given the current social distancing guidelines, licensing examinations, such as USMLE Step 2 Clinical Skills (CS), have been suspended through May 31, 2020, currently a minimum of 11 weeks [22]. Compounding this issue is that medical students taking this exam may not have had a full year’s preparation of clinical opportunities given the suspension in clinical clerkships. USMLE Step 2 Clinical Knowledge (CK) also faces extended suspension, and the scoring of this exam is used in evaluating candidates for residency [22-23]. As a result, in conjunction with USMLE Step 1 moving to pass/fail and the AAMC’s recommendation of waiving these requirements, it may be hard to discern among the various levels of qualified (or unqualified) applicants [24-25].

Mental health ramifications

Medical students are particularly vulnerable to the mental health ramifications of the COVID-19 pandemic. The global prevalence of anxiety and depression among medical students is 33.8% and 33% respectively, both substantially higher than the general population [26-27]. Social distancing policies and physical isolation may incite acute stress disorders, irritability, fear and panic, avoidance behavior, emotional distress, and other mental health consequences in the healthy population, but vulnerable groups, such as medical students, may be more at risk [28]. Studies of Chinese college students during the COVID-19 pandemic have shown positive correlations between academic delays and anxiety symptoms [29]. From, an academic perspective, mental health distress in at-risk medical students has the potential to negatively impact academic performance [10,30]. Therefore, what is the enduring impact the psychological ramifications of COVID-19 on residency applications? Poor academic performance, along with negative psychological impacts, as a result of COVID-19, may potentially hinder at-risk medical students as they apply to residency programs.

Implications for residency applications

The undeniable question moving forward is how the aforementioned challenges of the COVID-19 pandemic will affect medical students applying to residency in 2021. We have outlined how suspensions in clinical clerkships, changes in grading structures, and the lack of away rotations, and mental health concerns may negatively impact medical students’ applications to residency training programs. The National Residency Matching Program (NRMP) has acknowledged the COVID-19 pandemic’s effect on UME and is continuously evaluating changes that may need to be made for medical students graduating next year. Thus far, there are no plans to change the residency match schedules in 2021 [31].

Possible suggestions for residency applications

So how can residency training programs respond to such unprecedented changes in the clinical training of medical students in the time of COVID-19? Although the AAMC has not yet released general suggestions for the upcoming application cycle, some specialties have already started to adjust the residency application process. “The Council of Residency Directors in Emergency Medicine (CORD) Advising Students Committee in Emergency Medicine (ASC-EM) anticipates institutional and regional variability in both the spread and response to COVID-19” [32]. As a result, emergency medicine governing bodies have suggested reducing the requirement of standardized letters of evaluation (SLOE) and limiting the maximum number of away rotations [32]. On April 14, 2020, the Association of Professors of Gynecology and Obstetrics (APGO) with the Council on Resident Education in Obstetrics and Gynecology (CREOG) released a response to the COVID-19 pandemic’s impact on obstetrics and gynecology (OB/GYN) residency applications. by limiting the suggested number of away rotations for those students who will not have experiences locally and flexibility in the number of specialty-specific letters of recommendation. Additionally, both APGO and CREOG suggest postponing the OB/GYN application submission deadline and residency interviews to account for the pandemic’s impact on clinical experiences. Finally, these governing bodies suggest considering the opportunity for video interviewing for residency positions. The goal of these recommendations is to provide an equitable application process in the upcoming cycle amidst the disruption of the COVID-19 pandemic [33]. Similar future generalized recommendations for UME governing medical will best assure uniformity and equitable opportunity for students applying to all specialties in the upcoming cycle.

## Conclusions

The current COVID-19 pandemic has created global challenges in medical education. The academic risk posed to current UME trainees is great as clinical rotations have been suspended and many classes moved to a virtual platform. Historical precedents, like Hurricane Katrina and the SARS outbreak, have provided a foundation as to how medical education can adequately be adjusted in times of crisis, while also highlighting that there will be lasting impacts on academic performance, education, and residency applications. The current situation necessitates adaptability on behalf of students, medical schools, residency programs, and accrediting bodies. A conscious view of how decisions will affect medical trainees is necessary to ensure equality of education and proper preparation of learners in their application to residency training programs.
